# Effects of bacteriophage supplement on the growth performance, microbial population, and *PGC-1α* and *TLR4* gene expressions of broiler chickens

**DOI:** 10.1038/s41598-022-18663-1

**Published:** 2022-08-23

**Authors:** Zahra Sarrami, Mohammad Sedghi, Ishmael Mohammadi, Woo Kyun Kim, Amir Hossein Mahdavi

**Affiliations:** 1 Department of Animal Sciences, College of Agriculture, Isfahan University of Technology, Isfahan, 84156-83111 Iran; 2 Department of Poultry Science, University of Georgia, Athens, GA USA

**Keywords:** Microbiology, Physiology

## Abstract

Bacteriophages (BP) are viruses that invade bacteria and propagate inside them, leading to the lysis of the bacterial cells. The aim of this study was to investigate the effect of adding BP to the broiler’s diet and its effect on the performance, morphology and bacterial population of the gut, some immune responses and expression of some intestinal genes. Accordingly, dietary treatments were as follows: basal diet (control), and control + 0.3 g/kg colistin or 0.5, 1 and 1.5 g BP/kg of diet. BP increased the body weight gain and reduced the feed conversion ratio (FCR), as compared to the colistin treatment, in the finisher and overall period (*P* < *0.05*). European efficiency factor was significantly higher in 1.5 g BP-fed birds, as compared to the control and colistin treatments. meanwhile, bacteriophage and colistin-fed birds had higher *Lactobacillus* and lowered coliform bacteria counts, as compared to the control treatment (*P* < *0.05*). Cecal concentrations of propionate in the 1.5 g BP-fed birds were higher than those in the control treatment (*P* < *0.05*). BP-fed birds had a significantly increased villus height to crypt depth ratio, as compared to the control treatment. BP increased the serum concentrations of the total antibody, immunoglobulin (Ig) M, and IgG, as compared to the control treatment (*P* < *0.05*). In the ileum, the expression of the Peroxisome proliferator-activated receptor gamma coactivator 1-alpha (*PGC-1α*) gene was decreased by dietary BP supplementation (*P* < *0.05*). Furthermore, Toll-like receptor 4 (*TLR4*) gene expression was down-regulated in the BP-fed birds, whereas Interleukin 10 (*IL-10*) gene expression was up-regulated (*P* < *0.05*). Overall, the use of BP may be a promising alternative to growth-promoting antibiotics in broilers by altering the gastrointestinal tract microbiota, enhancing immunological responses and improving the gut's morphology.

## Introduction

The intestinal microbiota is an important part of the gastrointestinal tract (GIT); it plays a vital role in determining the physiology, nutrient digestion, intestinal morphology and immune system, and protecting the host against pathogens^1^. Colonization of beneficial microbiota in the GIT prevents the growth of pathogenic bacteria, stimulates and develops the host’s immune system, and provides metabolic substrates needed by the birds (short-chain fatty acids (SCFA), vitamins, etc.)^2,3^. Various studies have reported that lactic acid-producing bacteria (*Lactobacillus*) make up 95% of the intestinal bacteria, with well-known effects on the function and health of the bird’s GIT^4^. Reducing the number of pathogenic bacteria is a promising strategy to increase beneficial bacteria, which could be due to the less competition with pathogenic bacteria for gut binding sites and nutrients^2^. Therefore, reducing pathogenic bacteria such as *E. coli* and *Salmonella* provides an opportunity for commensal bacteria (such as *Lactobacillus*) to grow and increase in a given population.

Antibiotics have been used since the 1940s to treat both humans and animals with various bacterial diseases. The effects of antibiotics as growth promoters on the animals’ performance was first reported by Moore et al.; they observed that birds fed with streptomycin exhibited higher growth responses^5^. In-feed antibiotics have beneficial effects on the host, such as improving the growth rate, reducing mortality and increasing the resistance to bacterial diseases. However, the use of in-feed antibiotics is associated with antibiotic resistance development, which is a public health threat^6^. In veterinary medicine, the antibiotic colistin has been widely administered to prevent and treat the diseases caused by *E. coli* and *Salmonella*^7^. Also, in countries where this practice is allowed, colistin is used in low doses as a growth promoter in poultry diets^8^. The use of colistin causes bacterial resistance, which can be transmitted from livestock to humans^9^. As such, the use of colistin and many other antibiotics for growth-promoting purposes in food animal production has been banned in several countries, thus encouraging researchers to identify safe alternatives such as probiotics, prebiotics, phytogenic and bacteriophages (BP)^10^. In this study, we are trying to investigate whether BP can be a substitute for growth-promoting antibiotics. Therefore, we compared the antibiotic colistin with BP because this antibiotic has a growth-stimulating effect at the sub-therapeutic level.

Bacteriophages were discovered in the early 1900s^11^. Bacteriophages can infect bacteria and propagate inside them, leading to the lysis of the bacterial cells^12^. Generally, BP is intracellular parasites propagated inside bacteria by using the host biological organs^13^. Bacteriophages’ infection begins when they attach themselves to the surface receptors of the bacterium. To bind BP to bacteria, their tail must recognize the matching bacterial antigen. Therefore, they act specifically to infect bacteria, and most BP can infect only one or a limited number of bacterial species^14,15^. Each prophage follows a unique path after entering into the bacteria (lytic and lysogenic cycles). Some of them enter the lytic cycle, where BP insert its DNA or RNA into the bacterial genome. Then, the bacterial genome stops replicating. The prophage uses bacterial organelles and enzymes to replicate its genome to produce more BP, which are released after bacterial lysis. Therefore, lytic BP can result in rapid bacterial death^14,16^. Lytic BP is the best for therapeutic purposes in bacterial infections^17^. As such, BP can be used as a safe alternative to antibiotics because they have no detrimental effects on the eukaryotic cells^10^. Previous studies on BP have been mainly conducted under some bacterial challenge conditions, where BP has been used to treat active infections. Limited research has been conducted in regard to the effects of BP under a normal physiological state. Zhao et al., however, examined the effects of BP on the laying hens, reporting that adding 0.035% and 0.05% of BP to the layer diet significantly improved egg production^18^. In addition, it has been reported that dietary supplementation with BP could improve the growth performance of broilers^19^. However, more studies are needed to prove the efficacy of BP and its mode of action in the GIT of poultry.

The regulation of beneficial digestion in the GIT of chickens is dependent on the critical genes transcription of intestinal epithelial cells (IEC). *PPARγ* and *PGC-1α* are among important genes involved in the IEC metabolism, migration of enterocyte stem cells from crypts to villi and probably, regulation of intestinal inflammation. Also, the *TLR4* gene is responsible for producing the enterocyte cell's surface protein for pathogens recognition. In these conditions, IECs release the IL-10 cytokine to prevent inflammation. Therefore, the present study investigated the transcription of these genes in the ileal section through BP supplement inclusion in the broiler's diet. Furthermore, this study hypothesized that BP could have beneficial effects on the performance, immune responses, and microbial population in broilers reared with no bacterial challenge.

## Materials and methods

### Bird’s management and experimental design

This experiment was based on the comprehensive animal welfare guide (FASS, 2010). All experiments and stages of animal care were approved by the Animal Policy and Welfare Committee of Isfahan University of Technology. This study also followed the ARRIVE guidelines. The temperature of the rearing hall was 33 ℃ in the first two days and then gradually decreased to 22 ℃ at the age of 39 days. The bird’s light program was considered according to the protocols available in scientific sources; so the light regime was 23L: 1D for the first three days; then it was 20L: 4D until the end of the experiment. Broiler chickens were fed ad libitum with the standard diets (Table 1) during the starter (1–10 days), grower (11–24 days) and finisher periods (25–39 days).

**Table 1:** The composition and nutrient content of the control diet (g/kg).

Ingredients	Starter	Grower	Finisher
Corn	518.20	563.70	624.60
Soybean meal (CP = 42%)	370.00	349.00	281.00
Soybean oil	20.00	25.50	30.30
Corn gluten meal (CP = 60%)	50.00	25.00	30.00
Salt	2.10	2.20	1.90
NaHCO_3_	2.30	2.20	2.60
Di-calcium phosphate	16.50	13.80	12.20
Limestone	10.40	9.70	8.70
Vitamin premix^a^	1.00	1.00	1.00
Mineral premix^b^	1.00	1.00	1.00
l-Lysine-HCL	3.40	2.50	2.70
dl-Methionine	3.00	2.80	2.60
l-Threonine	1.00	0.70	0.50
Choline chloride	1.00	0.80	0.80
Phytase 5000 (FTU/g)	0.10	0.10	0.10
**Analyzed compositions**			
Metabolisable energy (Kcal/kg)	2985.00	3040.00	3155.00
Crude protein (%)	23.00	20.90	18.82
Digestible lysine (%)	1.28	1.15	1.02
Digestible methionine (%)	0.64	0.58	0.54
Digestible methionine + cysteine (%)	0.95	0.87	0.80
Digestible threonine (%)	0.86	0.77	0.68
Calcium (%)	0.96	0.87	0.78
Available phosphorus (%)	0.48	0.43	0.39

^a^Supplied per kg of diet: 12,000 IU Vit A, 5000 IU Vit D3, 80 IU Vit E, 3.2 mg Vit K, 3.2 mg Vit B1, 8.6 mg Vit B2, 65 mg niacin, 20 mg pantothenic acid, 4.3 mg Vit B6, 0.22 mg biotin, 2.2 mg folic acid and 0.017 mg VitB12.

^b^Supplied per kg of diet: 16 mg copper, 1.25 mg iodine, 20 mg iron, 120 mg manganese, 0.3 mg selenium and 110 mg zinc.

A total of 1200 1-day-old *as-hatch* broilers (Ross 308) with an average weight of 37.35 ± 0.82 g were used based on a completely randomized design with five treatments and ten replicates of 24 chickens. Dietary treatments included control diet (the basal diet without BP), control diet + 0.5 g BP/kg of diet; control diet + 1 g BP/kg of diet, control diet + 1.5 g BP/kg of diet, and control diet + 0.3 g colistin/kg of diet. ProBe-Bac (Pathway Intermediates, Seoul, South Korea) was used as a BP supplement. ProBe-Bac is a BP cocktail (a mixture of several BP) that has 2.04 × 10^8^ PFU/g BP against *Salmonella* and *E. coli*. The body weight of each chicken was recorded individually at 10, 24 and 39 days of age after two hours of not feeding. Also, feed intake was recorded at the end of the 10, 24 and 39 days of age. Then, the average daily weight gain, average daily feed intake, feed conversion ratio (FCR), and European efficiency factor (EEF) were calculated accordingly. In order to sample the collection, at the end of the experiment (39 days), one male chicken was selected randomly from each replicate and humanely euthanized by CO_2_.

### Intestinal sample collection for detecting ileal bacteria

At the end of the experiment, the ileal contents of six chickens from each treatment were used to enumerate lactobacillus and coliform bacteria (due to experimental limitations, we used six replicates). Briefly, 1 g of digesta was collected (from the last 10 cm of the ileum) and mixed with 9 mL of peptone water broth^20^. The solution was then homogenized and serially diluted (1:10) in phosphate-buffered saline. The population of *Lactobacillus* was counted on the MRS agar (Man, Rogosa, and Sharpe agar, Ibresco, Iran) and incubated in an anaerobic incubator at 37 °C for 48 h^21^. The coliform bacteria population was counted on the MacConkey agar (Ibresco, Iran) and then incubated at 37 °C for 24 h^22^. Microbial concentrations results were presented as a log10 colony-forming unit (CFU) per 1 g of ileal digesta.

### Analysis of cecal short-chain fatty acids (SCFAs)

On day 39 of their age, the cecal contents of ten euthanized birds from each treatment were collected and stored at − 20 °C. Cecal contents (1 g) were thawed and suspended in 4 mL of distilling water in a sterile tube. Samples were homogenized and centrifuged at 4000×*g* for 15 min. The supernatant (1 mL) was then transferred into an Eppendorf tube and mixed with a 250 µL metaphosphoric acid solution. The solution was centrifuged again at 4000×*g* for 15 min. Subsequently, 1 mL of the supernatant was transferred into an Eppendorf tube, mixed with 200 µL crotonic acid solution, and centrifuged at 4000×*g* for 15 min. Concentrations of acetate, propionate, butyrate, isobutyrate, valerate and isovalerate of the supernatant were determined by gas chromatography (Chrompack CP9002)^23^.

### Analysis of the immune system response

#### Weighing the lymphatic organs

At the end of the experiment, lymphoid organs such as the bursa of Fabricius, thymus and spleen were separated and weighed individually from ten euthanized chickens per treatment. The relative lymphoid organ weight was then calculated and presented as a percentage of the live body weight.

#### Anti-SRBC antibody assay

Ten birds from each treatment were injected with 0.5 mL of 5% suspension of sheep red blood cell (SRBC) on days 22 and 29 of age to evaluate the antibody production potential. Briefly, on day 22 of the experiment, one bird was randomly selected from each replicate and then injected with SRBC solution. Then, the bird was painted to distinguish it from other chickens in the pen. On the day 29 of the experiment, the same bird was injected with the SRBC again after blood sampling. One week after the second injection, blood sampling was performed from the injected bird. The serum of each sample was isolated and frozen at − 20 °C. Anti-SRBC antibody was measured using the microhemagglutination assay in 96-well microplates, as described by Akhlaghi et al.^24^.

#### Intestinal morphological analysis

**Figure 1:**
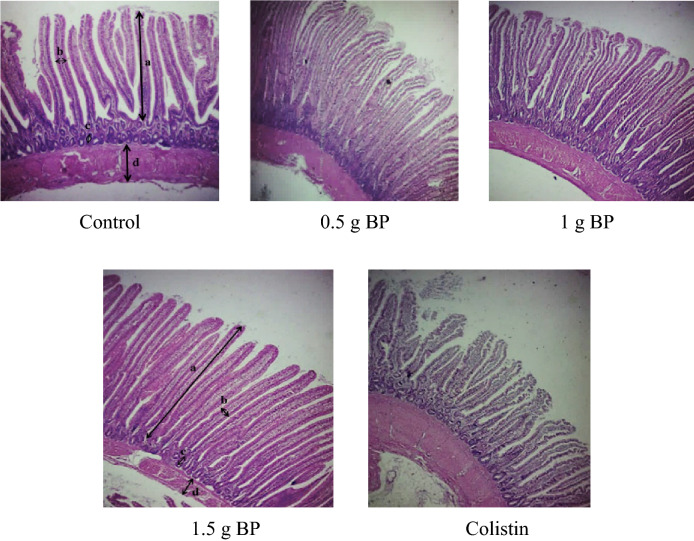
Effect of bacteriophage and colistin on the jejunal histological changes in the broilers at 39 d of age. 0.5 g BP: 0.5 g/kg bacteriophage in diet, 1 g BP: 1 g/kg bacteriophage in diet, and 1.5 g BP: 1.5 g/kg bacteriophage in diet. Arrows: a: villus height, b: villus width, c: crypt depth, and d: muscle thickness.

At the end of the experiment, jejunal samples were collected from ten euthanized birds per treatment. One cm of the jejunal's midpoint from each bird was removed and fixed in 10% buffered formalin. Samples were prepared, as described by Ekim et al., for evaluation by an optical microscope (Olympus CX31, Tokyo, Japan) and photographed with a digital microscope camera. Height and width of villi and crypt depth, and muscular layers’ thickness were determined, as shown in Fig. 1; they were measured using the ImageJ software^25,26^. The formula used for calculating the villus surface area was “2π × (average villus width/2) × villus height”^27^.

### Sample preparation and total RNA extraction

On day 39 of age, after euthanizing with CO_2_, two cm of the midpoint of the ileum of three chickens^28,29^. from each treatment was obtained for the RNA extraction. The collected tissue samples were washed with distilled water and immediately frozen in liquid nitrogen and stored at − 80 ℃. TRIzol (Sinaclon, Tehran, Iran) was then used for RNA extraction. The chicken intestinal tissue samples were mixed with liquid nitrogen in a sterile mortar and then crushed. After that, one ml of TRIzol was added to break the cells and vortexed intensely for 40 s. Subsequently, 200 μL of chloroform was added and centrifuged at 4 °C for 15 min at 12,000 rpm. The upper phase containing RNA was collected; then 500 μL of isopropanol was added and centrifuged at 11,000 rpm for 10 min. The upper phase was discarded and 1 mL of 75% ethanol was added to the RNA pellet and then centrifuged at 11,000 rpm. The upper phase was discarded at the end; after drying the pellets, 40 μL of the DEPC water was added to the RNA pellets. RNase-free DNase I was also used to remove the DNA contamination (Sinaclon, Tehran, Iran). The total RNA quantity and purity ratios (260/280 ratios) were calculated using a Nano Drop-2000 (Thermo, USA). The RNA extracted was stored at − 80 °C and used for future molecular analysis.

### Real-time quantitative RT-PCR (qRT-PCR) analysis

The expression of four candidate genes, including Peroxisome proliferator-activated receptor *γ* (PPARγ), *PGC-1α*, *TLR4* and *IL-10,* was determined using quantitative reverse transcription-PCR (QRT-PCR, ABI StepOne Real-Time PCR System—Thermo Fisher Scientific) to test the fold change of the selected genes. The reaction was performed using RealQ Plus 2× Master Mix Green (Amplicon). Primers were designed based on the target gene sequences and blasted with the NCBI Blast Primer; they were synthesized commercially (TAG Co. Copenhagen, Denmark) (Table 2). Complementary DNA (cDNA) was synthesized from the total RNA using cDNA Synthesis RT reagent Kit (Sinaclon). For each sample, 25 ng of cDNA was used as a template in the final volume of 25 μL reaction, which included 5 μl DEPC water, 2.5 μL forward and 2.5 μL reverse primers, 12.5 μl SYBR green containing Rox and 1 μL cDNA samples. The thermal cycling conditions included an initial denaturation step at 95 ℃ for 10 min, which was followed by 40 cycles including the denaturation step at 95 ℃ for 30 s, an annealing and extension step at 60 ℃ for 30 s, and elongation step at 72 ℃ for 30 s. Finally, the melt curve stage including 95 ℃ for 15 s, 60 ℃ for 60 s, and ramp speed at %100 and 95 ℃ for 15 s. For the interior control, the *glyceraldehyde 3-phosphate dehydrogenase* (GAPDH) gene was selected. All examinations were done in triple, successively and autonomously. The cycle threshold (Ct) values of the triplicate PCRs were averaged, and the relative quantification of the transcript levels was carried out by utilizing the comparative 2^−ΔΔCT^ method. The fold change in the target gene, relative to *GAPDH,* was determined according to the following formula: fold change = 2^−ΔΔCT^, where ΔCT = CT (a target gene) − CT (a reference gene), ΔΔCT = ΔCT (a target sample) − ΔCT (a reference sample); every sample was further fortified without inverse transcription to ensure that no DNA impurity would be in the sample.

**Table 2:** Specific amplification of the gene and internal reference primer.

Gene	Forward primer (5′–3′)	Reverse primer (5′–3′)	References
*GAPDH*	GAAGCTTACTGGAATGGCTTTCC	CGGCAGGTCAGGTCAACAA	^30^
*PPARγ*	CACTGCAGGAACAGAACAAAGAA	TCCACAGAGCGAAACTGACATC	^31^
*PGC1-α*	GACACAACACGGACAGAACT	GCATCACAGGTATAACGGTAGG	^32^
*TLR4*	AGTCTGAAATTGCTGAGCTCAAAT	GCGACGTTAAGCCATGGAAG	^33^
*IL-10*	CTGTCACCGCTTCTTCACCT	ACTCCCCCATGGCTTTGTA	^34^

### Statistical analyses

Data were analyzed using the GLM procedures of SAS 9.4 statistical software (SAS Institute, 2009), as a completely randomized design (CRD). Also, the normality procedure was used to check the normality of the data. Tukey’s tests were also used to determine the difference between treatments. Values were considered statistically different at *P* < *0.05*. Trends (*0.05* ≤ *P* ≤ *0.1*) were also presented. Additionally, the linear and quadratic effects of the supplemental BP were analyzed using polynomial contrasts. Orthogonal contrast was also analyzed in SAS 9.4 (2009) to test control versus BP-supplemented birds.

### Ethics approval

#### Animal welfare statement

The authors confirm that they have adhered to the animal welfare statement’ in this manuscript, and they confirm that all of the EU standards for the protection of animals and/or feed legislation have been met. The only exception was for stock density; in this case, the final body weight was set to be less than 30 kg/m^2^, which was lower than that mentioned in the council directive 2007/43/EC of June 28, 2007. We also confirm that we have followed the animal welfare guide, as adopted by FASS (2010). All animal care and experimental procedures were approved by the animal policy and welfare committee of Isfahan University of Technology. Also, this study followed the ARRIVE guidelines.

## Results

### Growth performance

The results related to the performance of broilers during the entire experimental period are shown in Table 3. The mortality rate was low (less than 5% per treatment) during the experiment period; therefore, the mortality rate per treatment was not analyzed. The inclusion of colistin and BP did not affect the body weight gain and FCR during the starter and grower periods. Feed intake was only affected by dietary treatments in the grower period; the lowest and highest feed intake was observed in the colistin and 0.5 g/kg BP fed groups (*P* < *0.05*), respectively. Dietary inclusion of BP increased the body weight gain and EEF, but it reduced the FCR, when compared to the control and colistin-fed groups (*P* < *0.05*) during the finisher and overall period (*P* < *0.05*). In addition, the EEF in the birds fed with 1.5 g BP was significantly higher than in those fed control or colistin-containing diets (*P* < *0.05*). Overall, adding BP to the diet linearly improved the body weight, FCR and EEF, whereas adding colistin caused a drop in the performance and EEF.

**Table 3:** Effect of adding BP to the diet on performance during the experimental periods.

Item	Dietary treatments						P-value^5^			
	Control	0.5 g BP	1 g BP	1.5 g BP	Colistin	SEM	Trt	C vs BP	Lin	Quad
**0–10 days**										
ADWG^1^ (g)	21.20	21.53	21.61	21.60	21.58	0.28	0.830	0.255	0.318	0.561
ADFI^2^ (g)	24.56	23.90	24.23	24.36	24.26	0.25	0.473	0.162	0.804	0.110
FCR^3^	1.16	1.11	1.12	1.12	1.12	0.01	0.139	0.025	0.191	0.076
**11–24 days**										
ADWG (g)	52.73	53.73	52.23	52.64	50.92	0.70	0.102	0.868	0.587	0.682
ADFI (g)	70.98^ab^	72.03^a^	69.11^ab^	70.50^ab^	68.40^b^	0.87	0.039	0.662	0.262	0.842
FCR	1.35	1.34	1.32	1.33	1.34	0.01	0.756	0.400	0.466	0.445
**25–39 days**										
ADWG (g)	88.92^ab^	92.11^a^	93.54^a^	92.53^a^	87.23^b^	1.17	0.001	0.006	0.020	0.071
ADFI (g)	146.21	148.84	147.67	146.64	145.24	1.70	0.625	0.391	0.981	0.233
FCR	1.64^ab^	1.61^ab^	1.58^b^	1.58^b^	1.66^a^	0.01	0.012	0.018	0.010	0.349
**0–39 days**										
ADWG (g)	58.56^ab^	60.24^a^	60.27^a^	60.03^a^	57.36^b^	0.56	0.001	0.009	0.059	0.067
ADFI (g)	88.01	89.23	87.82	87.95	86.63	0.79	0.272	0.694	0.621	0.449
FCR	1.50^ab^	1.48^abc^	1.45^c^	1.46^bc^	1.51^a^	0.01	0.001	0.002	0.002	0.120
EEF^4^	384.65^bc^	402.94^ab^	400.85^ab^	404.20^a^	379.92^c^	6.15	0.016	0.014	0.044	0.226

0.5 g BP: 0.5 g/kg bacteriophage in diet; 1 g BP: 1 g/kg bacteriophage in diet; 1.5 g BP: 1.5 g/kg bacteriophage in diet.

^1^Average daily weight gain.

^2^Average daily feed intake.

^3^Feed conversion ratio.

^4^European efficiency factor.

^5^Trt: Overall effects of treatments; C vs BP: contrasting birds not supplemented with BP with those supplemented with BP; Lin: linear effects of increasing the inclusion levels of BP; Quad: quadratic effects of increasing the inclusion levels of BP.

^abc^Values within a row followed by different superscripts are significantly different. *P* < 0.05; Tukey’s pairwise test.

### Ileal bacterial population

The results of the ileal *Lactobacillus* and Coliform bacteria enumeration are shown in Table 4. Birds fed with BP and colistin had higher *Lactobacillus* counts, when compared to the control birds (*P* < *0.05*). Also, the dietary inclusion of BP linearly increased *Lactobacillus* and reduced the coliform bacteria counts in the ileal contents, as compared to the control group (*P* < *0.05*).

**Table 4:** Effects of bacteriophage on the microbial population of the ileal contents (Log10 CFU/g).

Treatments	*Lactobacillus*	Coliform bacteria
Control	7.90^c^	6.56^a^
0.5 g BP	8.20^abc^	6.38^ab^
1 g BP	8.04^bc^	6.43^ab^
1.5 g BP	8.41^a^	6.29^b^
Colistin	8.25^ab^	6.33^b^
SEM	0.08	0.04
**P-value**^**1**^		
Trt	0.002	0.003
C vs BP	0.005	0.002
Lin	0.002	0.002
Quad	0.678	0.682

0.5 g BP: 0.5 g/kg bacteriophage in diet, 1 g BP: 1 g/kg bacteriophage in diet, and 1.5 g BP: 1.5 g/kg bacteriophage in diet.

Trt: Overall effects of treatments; C vs BP: contrasting birds not supplemented with BP with those supplemented with it; Lin: linear effects of increasing the inclusion levels of BP; Quad: quadratic effects of increasing the inclusion levels of BP.

^abc^Values within a column followed by different superscripts are significantly different. *P* < 0.05; Tukey’s pairwise test.

### Cecal short-chain fatty acids

Results of the cecum fatty acids analysis are shown in Table 5. The cecal concentration of propionate linearly increased with the addition of BP to the diet. Furthermore, BP resulted in a significantly higher cecal concentration of total short-chain fatty acids when compared to the control birds. Dietary treatments did not, however, affect the cecal concentrations of acetate, butyrate, isobutyrate, valerate, isovalerate, and acetate to propionate ratio.

**Table 5:** Effect of adding bacteriophage to the diets on the concentration of SCFAs in the cecum (mmol/g).

Treatments	Acetate	Propionate	A:P^1^	Butyrate	Isobutyrate	Valerate	Isovalerate	Total FA
Control	42.31	4.64^b^	10.02	7.12	0.20	0.49	0.54	55.32
0.5 g BP	50.13	6.03^ab^	8.78	8.39	0.22	0.49	0.63	65.92
1 g BP	52.18	6.10^ab^	9.24	8.85	0.24	0.57	0.69	68.68
1.5 g BP	51.71	8.03^a^	7.23	9.09	0.28	0.57	0.70	70.42
Colistin	49.57	6.14^ab^	8.58	6.33	0.23	0.51	0.57	63.38
SEM	4.16	0.59	1.16	1.19	0.05	0.09	0.10	5.05
**P-value**^**2**^								
Trt	0.462	0.006	0.551	0.454	0.840	0.934	0.740	0.264
C vs BP	0.085	0.004	0.267	0.273	0.425	0.592	0.208	0.043
Lin	0.134	0.0001	0.160	0.288	0.238	0.424	0.197	0.054
Quad	0.354	0.656	0.755	0.659	0.821	0.991	0.653	0.411

0.5 g BP: 0.5 g/kg bacteriophage in diet; 1 g BP: 1 g/kg bacteriophage in diet; 1.5 g BP: 1.5 g/kg bacteriophage in diet.

^1^Acetate to propionate ratio.

^2^Trt: Overall effects of treatments; C vs BP: contrasting birds not supplemented with BP with those supplemented with it; Lin: linear effects of increasing the inclusion levels of BP; Quad: quadratic effects of increasing the inclusion levels of BP.

^ab^Values within a column followed by different superscripts are significantly different. *P* < 0.05; Tukey’s pairwise test.

### Jejunal histological changes

Results of the jejunal histological analysis in the broilers are shown in Table 6. The addition of BP linearly increased the villus height and reduced the crypt depth, consequently increasing the villus height to crypt depth ratio and villus surface area (*P* < *0.05*). Meanwhile, adding colistin to the diet resulted in a significantly lower villus height and villus surface area, as compared to the 1.5 g BP fed group. No differences were, however, found in the villus width and muscle thickness among the treatments. Histological changes in the intestinal morphology with increasing the BP levels in the diet are presented in Fig. 1. Chickens fed with 1 and 1.5 g of BP/kg had a taller and more regular villus than the control and colistin treatments.

**Table 6:** Effect of adding bacteriophage to the diets on the jejunal histological changes.

Treatments	VH^1^ (µm)	CD^2^ (µm)	VH:CD^3^	VW^4^ (µm)	VSA^5^ (mm^2^)	MT^6^ (µm)
Control	983.51^ab^	127.09^a^	7.83^c^	90.97	0.28^ab^	285.53
0.5 g BP	1001.95^ab^	104.37^b^	9.68^b^	92.31	0.29^ab^	286.40
1 g BP	1000.10^ab^	98.58^b^	10.17^ab^	96.13	0.30^ab^	285.40
1.5 g BP	1133.46^a^	97.91^b^	11.57^a^	96.95	0.34^a^	287.66
Colistin	904.73^b^	103.93^b^	8.72^bc^	95.73	0.27^b^	281.27
SEM	41.82	13.48	0.40	3.27	0.01	13.28
**P-value**^**7**^						
Trt	0.008	< 0.0001	< 0.0001	0.637	0.032	0.997
C vs BP	0.238	< 0.0001	< 0.0001	0.281	0.170	0.946
Lin	0.030	< 0.0001	< 0.0001	0.148	0.018	0.921
Quad	0.205	0.002	0.620	0.937	0.298	0.955

0.5 g BP: 0.5 g/kg bacteriophage in diet; 1 g BP: 1 g/kg bacteriophage in diet; 1.5 g BP: 1.5 g/kg bacteriophage in diet.

^1^Villus height.

^2^Crypt depth.

^3^Villus height to crypt depth ratio.

^4^Villus width.

^5^Villus surface area.

^6^Muscle thickness.

^7^Trt: Overall effects of treatments; C vs BP: contrasting birds not supplemented with BP with those supplemented with it; Lin: linear effects of increasing the inclusion levels of BP; Quad: quadratic effects of increasing the inclusion levels of BP.

^abc^Values within a column followed by different superscripts are significantly different. *P* < 0.05; Tukey’s pairwise test.

### Immune system responses

Antibody responses, such as total, immunoglobulin (Ig) G and IgM levels, to anti-SRBC administration were measured (Table 7). In the primary response (29 d), the production of IgM tended to increase in the birds fed with BP, as compared to the control birds (*P* = *0.071*). In the secondary response (36 d), the BP-fed chickens had greater total anti-SRBC and IgG antibody titers in comparison to the control birds (*P* < *0.05*). No differences were found in the antibody titers between BP and colistin birds in terms of the primary and secondary responses. The addition of BP linearly increased the weight of the thymus (*P* = *0.003*) and bursa of Fabricius (*P* = *0.033*), whereas it tended to linearly increase the weight of the spleen (*P* = *0.067*).

**Table 7:** Effect of bacteriophage on the response to SRBC and weight of lymphoid organs.

Treatments	Immunoglobulin titers (Log 2)						Weight of lymphoid organs (% of live BW)		
	Primary response (29 days)			Secondary response (36 days)					
	Total	IgG	IgM	Total	IgG	IgM	Spleen	Bursa of Fabricius	Thymus
Control	3.20	1.90	1.30	4.20^b^	2.70	1.60	0.09	0.14^b^	0.34^b^
0.5 g BP	3.20	1.30	1.90	4.70^ab^	2.70	2.00	0.09	0.12^b^	0.38^ab^
1 g BP	4.10	2.20	1.90	6.10^ab^	3.90	2.20	0.10	0.14^ab^	0.42^a^
1.5 g BP	4.30	2.20	2.10	6.20^a^	3.90	2.30	0.10	0.18^a^	0.38^ab^
Colistin	3.70	2.20	1.40	5.90^ab^	3.60	2.40	0.10	0.16^ab^	0.35^b^
SEM	0.51	0.39	0.31	0.48	0.38	0.40	0.004	0.008	0.01
**P-value**^**1**^									
Trt	0.442	0.439	0.317	0.014	0.051	0.650	0.225	0.001	0.008
C vs BP	0.261	1.000	0.071	0.005	0.071	0.221	0.050	0.484	0.005
Lin	0.071	0.339	0.092	0.000	0.006	0.200	0.067	0.003	0.033
Quad	0.844	0.475	0.524	0.643	1.000	0.706	0.339	0.007	0.011

0.5 g BP: 0.5 g/kg bacteriophage in diet, 1 g BP: 1 g/kg bacteriophage in diet and 1.5 g BP: 1.5 g/kg bacteriophage in diet.

^1^Trt: Overall effects of treatments; C vs BP: contrasting birds not supplemented with BP with those supplemented with it; Lin: linear effects of increasing the inclusion levels of BP; Quad: quadratic effects of increasing the inclusion levels of BP.

^ab^Values within a column followed by different superscripts are significantly different. *P* < 0.05; Tukey’s pairwise test.

### Gene expression

**Figure 2:**
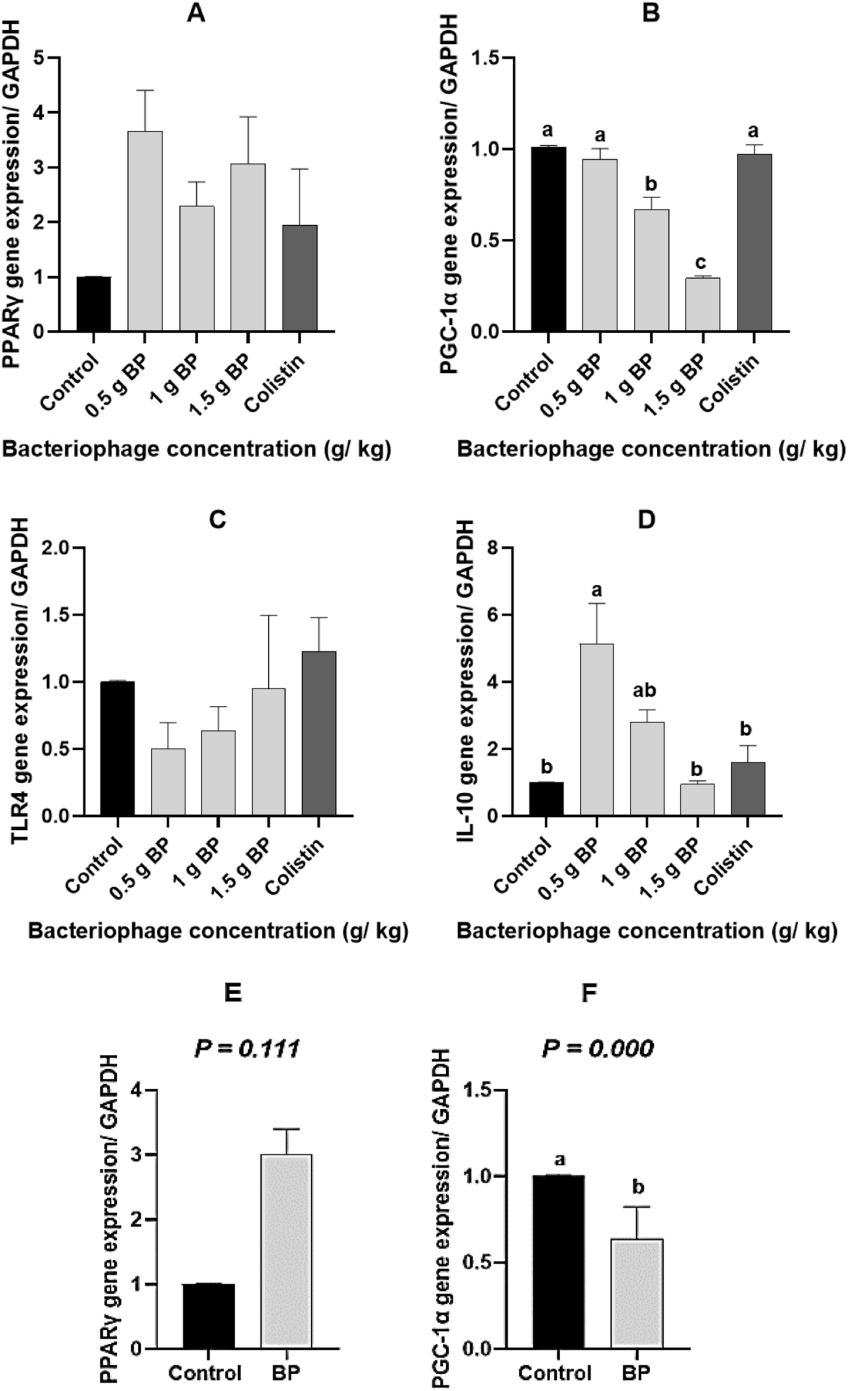

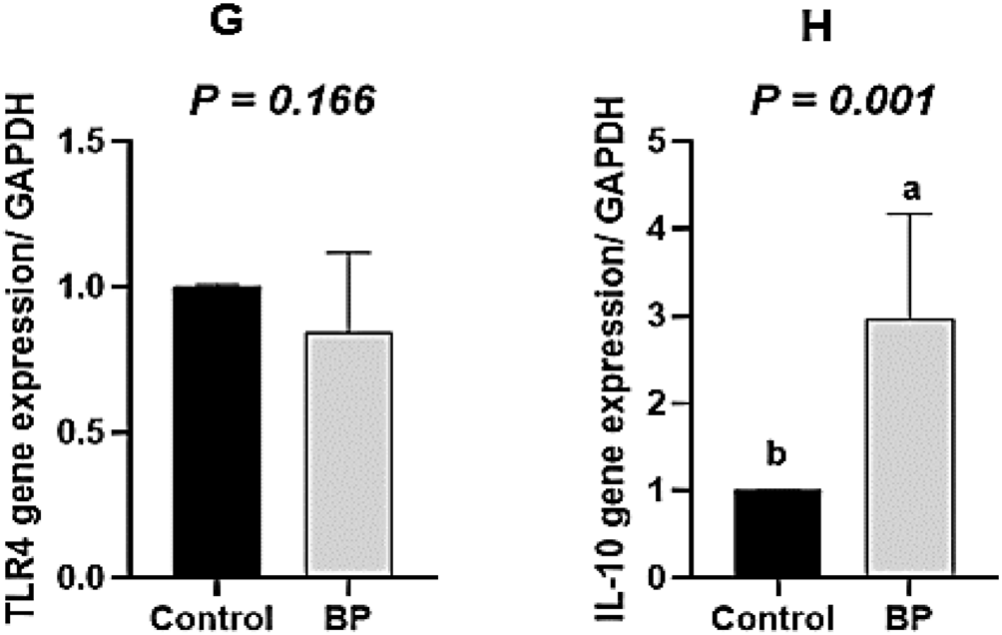
The expression of genes involved in inflammation and metabolism in the ileal tissue of broiler chicks: (**A**) *PPARγ* gene expression, (**B**) *PGC-1α* gene expression, (**C**) *TLR4* gene expression, and (**D**) *IL-10* gene expression, (**E**–**H**) Contrast analysis of *PPARγ*, *PGC-1α*, *TLR4* and *IL-10* genes expression. 0.5 g BP: 0.5 g/kg bacteriophage in diet, 1 g BP: 1 g/kg bacteriophage in diet, and 1.5 g BP: 1.5 g/kg bacteriophage in diet. ^abc^Values within a column followed by different superscripts are significantly different. *P* < 0.05; Tukey’s pairwise test.

The expression of the candidate genes is shown in Fig. 2. Treatments did not affect the *PPARγ* gene expression when compared to the control treatment (*P* > *0.05*; Fig. 2A). Feeding birds with 1 and 1.5 g of BP/kg of the diet led to the lower expression of the *PGC-1α* gene, as compared to the other birds (*P* < *0.05*; Fig. 2B). *IL-10* mRNA in the 0.5 g BP/kg treatment was higher than that in the other treatments (*P* < *0.05*). Furthermore, birds with 1 and 1.5 g BP/kg of the diet and colistin did not differ in the *IL-10* gene expression, as compared to the control-fed birds (*P* < *0.05*; Fig. 2D). Also, the difference in *TLR4* gene expression among the treatments was not significant. The orthogonal contrasts also showed that the mRNA expression of *IL-10 (P* = *0.001)* was elevated in the BP group, whereas *PGC-1α (P* = *0.0001)* was decreased with adding BP, as compared to the control diet, Also, the transcription of *TLR4* was not affected by any treatments (Fig. 2E–H).

## Discussion

In this study, adding BP to the diet of the broilers reared under normal physiological conditions (without bacterial challenge) improved their body weight gain, FCR and EEF. Therefore, BP could be considered as a candidate growth promoter to be included in the broiler diets. These results are, thus, in agreement with those reported by Kim et al., where body weight gain and FCR were improved by increasing the BP levels in the broilers’ diet^19,35^. A similar improvement in the layer's performance has also been observed in the previous studies^17,36^. However, Wang et al. found that no the BP supplementation had no beneficial effect on the body weight and FCR of broiler chickens^37^. Moreover, in a recent study, dietary BP supplementation was found to improve the performance of broilers and layers, while it reduced the concentration of *E. coli* and *Salmonella* in the excreta^38,39^.

It has been demonstrated that the BP therapy causes less change in the normal microflora of the GIT, as compared to growth-promoting antibiotics, which can often cause damage to the normal microbiota; such changes can lead to secondary infections^40^. Balancing the microbial population of the GIT increases the performance of the birds because it is generally accepted that the colonization of the beneficial microbiota in the GIT prevents the growth of pathogenic bacteria, stimulates and develops the immune system, as well as providing metabolic substrates needed by the birds (vitamins, SCFAs, etc.); thus, the gut microflora plays an essential role in utilizing and digesting nutrients^41^. In the present study, we used a BP cocktail (a mixture of several BP) that targeted *Salmonella* and *E. coli* bacteria. According to the results of the microbial culture in this study, the addition of BP to the diet reduced the population of coliform bacteria in the gut. As most of the coliform bacteria are pathogens, it can be concluded that the addition of BP to the diet may reduce pathogenic bacteria. In agreement with our results, Kim et al. (2014) reported that the addition of phage to the diet reduced the population of *Salmonella* and *Clostridium perfringens* in the cecal^35^. In addition, due to the increase in the population of *Lactobacillus* in the ileum of BP-fed birds, it could be concluded that the reduction of coliform bacteria and pathogens provided the conditions for the growth of beneficial *Lactobacillus* bacteria, and their population could be increased.

According to the results of the current study, birds fed with BP had a higher concentration of propionate in their cecum. The cecum is the location of the GIT with the highest concentration of SCFA. Although not all absorbed SCFAs enter the circulation, they can be used as an energy source in the epithelial cells^42^. Also, increasing the SCFAs concentration in the bird’s cecum reduces the gut pH, thus preventing the growth and proliferation of acid-sensitive pathogenic bacteria, such as the *Enterobacteriaceae* family^2^. In an in vivo study, a negative correlation was observed between the concentration of SCFAs (particularly acetate, propionate and butyrate) and the population of the *Enterobacteriaceae* family in the birds’ digestive tract^43^. Generally, SCFAs have a specific role in the GIT; these include producing energy by the gluconeogenesis process, reducing the harmful bacterial species in the GIT, stimulating the intestinal epithelial cells’ proliferation and maintenance of normal intestinal barrier functions, increasing the villus height and villus surface area, regulating the blood flow, stimulating the growth and proliferation of enterocytes, and controlling the mucin production, thus affecting the intestinal immune response^1,3^.

Intestinal morphology is a good indicator of the GIT health status and its response to the use of certain foods. The rapid maturation and development of the GIT can provide a good place for bacteria to colonize, and an increase of beneficial bacteria also leads to more development and growth of the GIT^44^. Improvement of the intestinal morphology, such as increased villus height and villus height to crypt depth ratio, improves bird's performance by enhancing the digestion and absorption of nutrients. Some dietary additives such as BP, probiotics and prebiotics can indirectly affect gut's morphology by manipulating the intestinal microbiota ecology. Reducing pathogenic bacteria and balancing the gut microbiota can affect the intestinal morphology and performance in several ways^3^. For example, some pathogenic bacteria, such as *E. coli*, can impair the digestive enzymes' secretion in two ways: 1. secreting proteolytic enzymes, which degrade the digestive enzymes, and 2. damaging the intestinal villus and microvillus^2^. Also, the activity of digestive enzymes is affected by the development of the GIT and intestinal microflora. For instance, *Bifidobacterium* and *Lactobacillus* increase the activity of digestive enzymes such as proteases, trypsin and lipases^1,45^. Therefore, the improved growth in the birds receiving BP could be due to the improved microbiota ecology, intestinal structure, increased villus height and width, and enhanced villus surface area.

In this study, feeding birds with colistin shortened the intestinal villus. Lei et al. also used virginiamycin antibiotic in the diet; they reported that the villus height in the duodenum and jejunum was shorter than that found in the control treatment^46^. According to another study, using antibiotics under normal conditions may have detrimental effects on the intestinal cells because they might reduce the normal bacterial population in the gut^42^. In this study, the birds were kept in hygienic conditions, with little microbial stress and contamination; therefore, colistin might have exerted detrimental effects on the intestinal cells due to the insufficient bacterial population in the gut, as it was evident by a shorter villus height. Additionally, according to a study conducted by Wang et al., the use of high doses of colistin could lead to the loss of tight junction in the gut; this is because the tight junction proteins' expression is reduced, thus destroying the intestinal mucosal barrier (in that study, 200 mg/kg of colistin was used in mice)^47^. This induces stress in the GIT and prevents the villi's growth^2^. Therefore, the dietary inclusion of growth-promoting antibiotics does not always result in a better performance of birds^41^.

The use of BP may be associated with the risk of immunological reactions. Although bacteria are a typical host for BP, some studies have shown that BP could also interact with some animal cells, especially with the immune cells^15,48^. Bacteriophages can enter into circulation regardless of the way they are administered. Some researchers have suggested that BPs enter the circulation within 2–4 h after ingestion; they are found in the internal organs (liver, spleen, etc.) approximately 10 h after ingestion^14^. When BP enter the circulation, they are eliminated very quickly from the inner organs and blood by phagocytic cells if they cannot find a bacterial host. Moreover, BP are also cleared by the cells of the reticuloendothelial system in the liver and spleen^49^. These findings, thus, suggest that the use of BP can activate the innate immune response. The host immune system produces neutralizing antibodies against BP^50,51^, along with non-neutralizing antibodies (IgM and IgG) in response to them^15,48^. In the current study, we found that as the level of BP was raised in the diet, serum concentrations of IgG and IgM were increased, which could strengthen the birds’ immune system. In addition, based on the cross-reactivity, neutralizing antibodies produced against phage may be able to affect other antigens that have similar structural areas. Cross-reactivity between antigens occurs when an antibody produced against a particular antigen tends to bind to a different antigen. This happens when antigens have similar structural regions and the antibody detects those^52^. Therefore, it may be concluded that antibodies produced against phage can prepare the body for a response to other similar viruses and help the immune system to respond quickly to viruses with similar structures. Overall, BP might strengthen the immune system in two ways: directly via entering the circulation system and stimulating the humoral and cellular immunological responses, and indirectly through their modulatory effects on the gastrointestinal microbiota.

In the present study, the relative weight of the lymphatic organs was measured as an indicator of the health status of the birds. In chickens, the size and weight of the bursa of Fabricius can provide general information regarding the maturation and development of the immune system because the bursa of Fabricius is a site for the maturation of T and B lymphocytes^53^. Generally, when the ratio of the bursa of Fabricius to body weight is about 0.2%, the birds are thought to have a functional immune system^54^. The thymus is a site for the maturation of T lymphocytes. Therefore, the increase in the thymus and bursa of Fabricius weights in response to the dietary inclusion of BP could indicate the development of the bird's immune system.

*The PPARγ* gene plays a key role in determining the health and inflammation conditions of the intestinal epithelial cells (IECs)^55^. The high expression of *PPARγ* in the intestine epithelial cells was approved. The *PPARγ*-dependent mechanism is necessary for anti-inflammatory responses during GIT inflammation in the Treg cells population in the lamina propria^56^. *PGC-1α* is a co-activator of *PPARγ*. However, the fact that *PPARγ* is modulated by lipopolysaccharides (LPS) suggests that *PPARγ* is involved in the LPS pathway^57^. In the chickens, *PPARγ* expression has one of the lowest transcripts in the adipose tissue. However, in the chicken's abdominal adipose tissue, *PPARγ* mRNA expression tended to increase with age^58^. The role of *PPARγ* in adipose tissue differentiation is probably mediated by co-activators^59,60^. *PGC-1α* is such a co-activator^61^. It has been identified that PPARγ serves a crucial role in the immune response via its ability to prevent the expression of inflammatory cytokines and facilitate the differentiation of the immune cells to the anti-inflammatory phenotypes. A characteristic of *PPARγ* is the constructive variety of its ligands, including endogenous metabolites and food components^62^. Since the presence of harmful bacteria in the GIT may lead to some inflammatory responses and increased mortality in birds, *PPARγ* agonists that naturally exist in the diets may be helpful to the poultry industry by inducing the production of the anti-inflammatory factors. Also, *PPARγ* serves a vital role in increasing the immune response via preventing the expression of inflammatory cytokines and navigating the differentiation of the immune cells to the anti-inflammatory phenotypes^62^. Previous studies have also demonstrated that *PPARγ* could considerably decrease the transcription of pre-inflammatory cytokine and down-regulate the inflammatory signaling pathways^63,64^. Although our results showed that the *PPARγ* gene expression was similar among birds, the dietary inclusion of BP numerically increased the *PPARγ* gene expression when compared to the control and colistin groups.

On the other hand, *PGC-1α* mRNA was significantly decreased in 1 and 1.5 g BP fed groups, which could decrease mitochondrial metabolism and intestinal cells’ signaling pathways and the factors involved in the cells’ apoptosis. *PGC-1α* is known to up-regulate mitochondrial biogenesis, respiratory capacity, oxidative phosphorylation, and fatty acid β-oxidation in various cell types. As described above, in successively regenerating intestinal epithelium, proliferative cells migrate from crypts to the villus axis; then they are differentiated into mature enterocytes. Based on the previous research, during cells migration and differentiation, the expression of *PPARγ* and *PGC-1α* is up-regulated; after a specific time, *PGC-1α* transcription will be down-regulated, leading to apoptosis^65^. It is also reported that the transcription of *PPARγ* and *PGC-1α* is not concurrent^66^.

Our results, therefore, demonstrate that the transcription of *PPARγ* and *PGC-1α* genes was not correlated in the ileal epithelial cells of the broiler chickens. In addition, Walter et al. demonstrated that the timing of upregulation of *PGC-1α *and *PPARγ* was not simultaneous^63^. Feeding birds with 1.5 g BP led to more developed jejunal cells, when compared to the other groups, as indicated by a taller villus height, the greater villus height to crypt depth and villus surface area. Therefore, it is likely that the 2–4-fold overexpression of *PPARγ* in the birds fed the diet containing of BP, through the specific downstream pathway, led to the differentiation of the stem cells in the intestinal crypts and the migration of these cells, leading to a more developed GIT.

In the present study, BP supplementation increased and decreased the population of *Lactobacillus* and coliform bacteria in the GIT, respectively, leading to the secretion of anti-inflammatory cytokines such as *IL-10*; this, in turn, reduced the expression of the *TLR4* inflammatory gene.

Previous studies have also shown that biliary secretion, feed intake, luminal bacteria, bacterial cell wall polymers, and host genetic sensitivity play substantial roles in regulating the pathogenesis effects on the small intestinal inflammation^67^. In the present study, the gene expression of *TLR4* was found to be numerically lower *(P* > *0.05)* in the birds fed with the BP-containing diet, as compared to the colistin group; however, it was similar in the control and 1.5 g BP fed birds. Meanwhile, the transcription of *IL-10* was increased in the birds fed 0.5 g BP/kg diet, in comparison to colistin and control groups. Naturally, bacteriophages act as a pathogens destroyers, decreasing LPS. Therefore, it resulted in the reduction of *TLR4* expression in the intestinal cells. This shows that bacteriophages potentially destroy the pathogenic bacteria in the GIT. Therefore, activation of the bacteriophage in the GIT diminishes its inflammation, resulting in the reduction of the *TLR4* gene expression.

The essential immune-regulatory function of *IL-10* is the inhibition of the effector functions of the activated phagocytes, T cells and non-immune cells. *IL-10* down-regulates the transcription and secretion of pro-inflammatory cytokines (*IL-1β*, *IL-6*, *IL-8*, *TNF-α*, and *G-CSF*) by the operated monocytes and macrophages^68,69^. This indicates that *IL-10* has different effects in various cell types. The inhibitory effect of *IL-10* on T cells may depend on the presence of activated macrophages or dendritic cells^70^, which can be activated by the presence of bacterial residues destroyed by bacteriophages. It has been demonstrated that *IL-10* knockout mice develop colitis, thus demonstrating that *IL-10* serves a key role in maintaining normal non-inflammatory intestinal immune-regulation^71^. In the present study, the gene expression of *IL-10* was increased by the BP supplementation at the level of 0.5 and 1 g/kg of the diet. Overall, these results suggest that bacteriophages can successfully modify the population of the intestinal bacteria, possibly leading to a healthy GIT, reduced intestinal inflammation, and improved differentiation and migration of proliferative cells in the intestinal crypts.

## Conclusions

While improving the intestinal health is not a new way to improve performance, it is crucial to find antibiotic alternatives in poultry farming systems where growth-promoting antibiotics are prohibited. The results of the current study showed that the application of BP under production conditions could be considered a promising alternative to growth-promoting antibiotics in broiler chickens. The present study also revealed that the orally administered BP could eliminate pathogenic bacteria, modulate the microbial composition of the GIT, and bring other benefits to the host, such as increasing the concentration of SCFAs, boosting the immune system, and improving the morphology of the intestine. We also found that some of these beneficial effects of bacteriophages might be modulated through the up-regulation of *IL-10* gene, which is involved in modifying the immune response of the GIT and activation of the downstream signaling pathways, thus improving epithelial cells metabolism through the up and down-regulation of *PPARγ* and *PGC-1α *genes, respectively. In general, this study suggests that supplementation of bacteriophage to the diet (at the level of 1–1.5 g/kg diet) is a good way to achieve the desired performance, metabolism and immune response.

## Data Availability

The datasets used and/or analyzed during the current study are available from the corresponding author upon reasonable requests.
